# Lead Poisoning Can Be Easily Misdiagnosed as Acute Porphyria and Nonspecific Abdominal Pain

**DOI:** 10.1155/2017/9050713

**Published:** 2017-05-29

**Authors:** Ming-Ta Tsai, Shi-Yu Huang, Shih-Yu Cheng

**Affiliations:** Department of Emergency Medicine, Kaohsiung Chang Gung Memorial Hospital, Chang Gung University College of Medicine, Kaohsiung 833, Taiwan

## Abstract

Lead poisoning (LP) is less commonly encountered in emergency departments (ED). However, lead exposure still occurs, and new sources of poisoning have emerged. LP often goes unrecognized due to a low index of suspicion and nonspecific symptoms. We present a case of a 48-year-old man who had recurring abdominal pain with anemia that was misdiagnosed. His condition was initially diagnosed as nonspecific abdominal pain and acute porphyria. Acute porphyria-like symptoms with a positive urine porphyrin test result led to the misdiagnosis; testing for heme precursors in urine is the key to the differential diagnosis between LP and acute porphyria. The final definitive diagnosis of lead toxicity was confirmed based on high blood lead levels after detailed medical history taking. The lead poisoning was caused by traditional Chinese herbal pills. The abdominal pain disappeared after a course of chelating treatment. The triad for the diagnosis of lead poisoning should be a history of medicine intake, anemia with basophilic stippling, and recurrent abdominal pain.

## 1. Introduction

Lead poisoning (LP) is rarely encountered in emergency departments (ED). However, occupational and nonoccupational exposures to lead occur worldwide [1]. Patients often present with nonspecific symptoms and signs such as abdominal pain, fatigue, anorexia, constipation, headache, irritability, and insomnia [2, 3]. Owing to the low incidence rate and nonspecific clinical features of LP, physicians seldom consider this rare disease entity in diagnosis. LP can cause significant morbidity and mortality if its diagnosis is delayed or incorrect and it is untreated. We report a case of a 48-year-old male patient with LP presenting as recurring abdominal pain and anemia that was initially misdiagnosed as nonspecific abdominal pain (NSAP) and acute porphyria.

## 2. Case Report

A 48-year-old man was admitted to our ED for a recurring abdominal pain that had developed over the previous month. He was a farmer who was admitted to a different hospital for the same reason without any significant improvement in his symptoms. Thus, the patient sought a referral to our hospital. On presentation at the ED, he was afebrile, with a blood pressure of 144/80 mmHg and pulse rate of 88 beats per minute. The abdominal pain was characterized as cramping and was generalized. Associated symptoms included poor appetite, nausea, abdominal distention, cold sweating, and general weakness. The pain was nonradiating and not accompanied by fever, diarrhea, or other symptoms. The abdominal examination showed hepatosplenomegaly and diffuse tenderness to deep palpation of the whole abdomen, without rebound tenderness and guarding. Systemic examinations revealed no abnormalities. The blood test conducted in our ED revealed hypochromic microcytic anemia with basophilic stippling of the erythrocytes, decreased hemoglobin concentration of 9.4 g/dL, and hematocrit level of 27.9%. Liver function test results were mildly abnormal. The white blood cell count and levels of creatinine, glucose, total bilirubin, lipase, and electrolytes were all within their normal limits. Abdominal radiographs and abdominal CT scan revealed no abnormalities. Liver echo, tumor marker, and viral hepatitis screening test results were negative. The patient was admitted to the internal medicine ward with a diagnosis of NSAP. The diagnosis of NSAP was made based on the clinical features and negative examination results. After admission, we performed a urine porphyrin test based on his acute porphyria-like symptoms. Acute intermittent porphyria was diagnosed based on a positive urine porphyrin test result, and hemin therapy was started. However, in spite of the hemin therapy, the pain did not improve, indicating a misdiagnosis. We performed a detailed medical history taking again. The patient reported visiting a local Chinese medicine clinic because of difficulty urinating and six herbal pills were prescribed to be taken daily for one month. It was noticed that the abdominal pain occurred after the intake of the Chinese herbal medicine. Therefore, the acute porphyria-like symptoms with a positive urine porphyrin test result, history of Chinese herbal medicine intake, and anemia with basophilic stippling raised the possibility of LP. To confirm whether LP caused the recurrent abdominal pain, we checked the urine lead level, urine delta-aminolevulinic acid (delta-ALA) level, urine porphobilinogen level, and blood lead level. The diagnosis was confirmed based on the high blood lead level of 62.8 *µ*g/dL (normal range, <40 *µ*g/dL) and urine lead level of 823.0 *µ*g/dL (normal range, <23 *µ*g/dL). The urine analysis revealed an elevated urine delta-ALA level of 81.8 mg/24 h (normal range, <7 mg/24 h) with a normal urine porphobilinogen level of 1.0 mg/24 h (normal range, <4 mg/24 h). The Chinese herbal pill was analyzed in the laboratory of the Department of Health and was found to contain an excessive amount of lead. Each pill was found to have up to 90 parts per million (ppm) lead, which is approximately 90 times higher than the maximum permissible limit for lead in certain food additives in Taiwan. The abnormal laboratory results obtained on admission and discharge are presented in Table 1. Based on the diagnosis of LP, the patient was treated with intravenous calcium disodium edetate (CaEDTA) at 1 g/d for 5 days as recommended by a toxicologist. On day 3 of chelation therapy, we noted a high body lead burden, as indicated by an increased urinary lead excretion up to 1853 *µ*g/dL/24 h. The clinical symptoms gradually improved. His blood lead level decreased to 31 *µ*g/dL, and his hemoglobin level and liver function had returned to normal levels when he was reevaluated 2 weeks after discharge. No relapse was observed thereafter and during the 1-year follow-up.

## 3. Discussion

LP can affect many systems in the body and consequently present with a wide range of symptoms, including fatigue, abdominal pain, headache, nausea, constipation, anemia, irritability, subtle mood changes, and pain in the hands, feet, muscles, or joints [3, 4]. These presentations can lead physicians to make a misdiagnosis of hematological, gastrointestinal, neuropsychiatric, cardiovascular, renal, or endocrine disorders [5–7].

As was evident in our case, abdominal pain is possibly the first and commonest manifestation of LP that leads to a visit to the ED. The abdominal pain in our patient was characterized as diffuse, severe, intermittent, and colicky pain. This resulted in a misdiagnosis of NSAP. A recent report described that the abdominal pain in LP is usually severe, intermittent, and poorly localized. It is sometimes associated with cramping (i.e., lead colic). It is also associated with gastrointestinal problems such as constipation, nausea, vomiting, diarrhea, and a sign of ileus such as abdominal distension and decreased bowel sounds [8]. LP should be considered as a differential diagnosis in cases of unexplained acute abdominal pain in the ED when other common causes have been excluded. In patients with unrecognized LP presenting with symptoms of abdominal pain, the condition can be easily misdiagnosed as acute cholecystitis, chronic pancreatitis, or appendicitis, and acute abdomen and gastrointestinal evaluation, as well as laparotomies, can be unnecessarily performed [9, 10]. The abdominal pain generally does not occur until lead levels are very high. The blood lead levels associated with abdominal pain are reported in the literature to range from 46 to >200 *µ*g/dL [11]. The initial lead level in our patient was 62.8 *µ*g/dL. At low blood levels (up to 10 *µ*g/dL), nonspecific symptoms are common, including malaise, anorexia, and irritability. Extremely high blood lead levels (>70 *µ*g/dL) could result in cerebral edema, encephalopathy with confusion, drowsiness, coma or seizures, and even death [11, 12]. The classical features of LP include abdominal pain (lead colic), anemia with basophilic stippling of red cells, blue-black gum deposits (Burton line), and lead line on joint radiography [11–13]. Making a diagnosis based on the classical clinical features such as abdominal pain and anemia is difficult because these are nonspecific features. Moreover, a lead line over the gum or joint often appears in chronic cases, but not in cases of acute poisoning such as in our patient.

Results of laboratory studies generally demonstrate hypochromic microcytic anemia, decreased liver function (AST/ALT levels), and elevated total and indirect bilirubin levels. The white blood cell count, renal function, and electrolyte levels are always within their normal limits [13]. Most patients undergo multiple hospital admissions, diagnostic studies, medication evaluations, and even laparotomies, all without benefit. Multiple studies of abdominal pain, such as endoscopy, ultrasonography, and abdominal CT yield negative results [3, 9, 11, 14]. Many different specialists such as surgeons, psychiatrists, gastroenterologists, neurologists, and emergency physicians may become variably involved in the diagnostic process, especially for cases presenting with acute and life-threatening clinical features [12, 14].

In our patient, the abdominal pain was induced by lead toxicity. He was hospitalized elsewhere and underwent extensive studies without any improvement. He had mild normocytic anemia and mildly elevated AST and ALT levels. Basophilic stippling of erythrocytes was found on blood film. However, no lead line over the gum or joint was found. These nonspecific findings did not lead us to consider LP as a differential diagnosis. In addition, the positive urine porphyrin test result raised the suspicion of acute intermittent porphyria and led us to administer hemin therapy without any benefit. A false-positive urine porphyrin test result is possible in porphyria induced by liver cancer, hepatitis, and heavy metal poisoning such as that with lead [15]. Liver cancer and hepatitis were not detected in our case during the examinations such as liver ultrasonogram and screening for tumor markers and viral hepatitis. Therefore, the acute porphyria-like symptoms with a positive urine porphyrin test result, anemia with basophilic stippling, and history of Chinese herbal medicine intake raised the possibility of LP.

**Figure 1:**
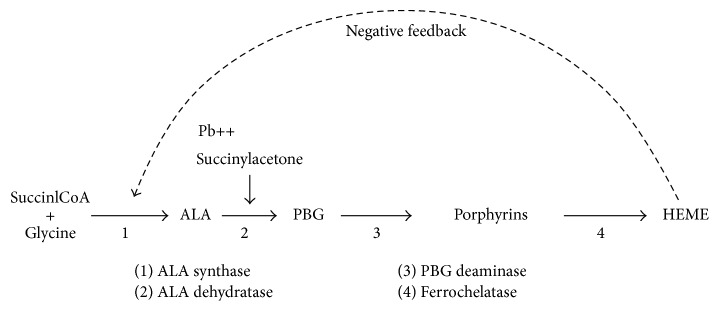
The heme synthetic pathway showing the enzymes mediating formation of delta-ALA, PBG, and the initial porphyrins (uroporphyrinogen), respectively. Lead poisoning and acute porphyria both affect the heme synthetic pathway. In LP, the metal directly inhibits the ALA dehydratase. The result is overproduction of delta-ALA only; PBG normal, reflecting the impaired conversion of delta-ALA to PBG. ALA = aminolevulinic acid; PBG = porphobilinogen; CoA = coenzyme A; Pb^++^ = lead.

LP and acute porphyria are etiologies of abdominal pain in ED. Both clinical features often resemble and can overlap, making it difficult to make a differential diagnosis based on clinical features alone. LP and acute porphyria both affect the heme synthetic pathway leading to a potentially limited heme production [16]. Acute porphyria, characterized by a potent, variable, catalytic defect of enzymes involved in the heme pathway, is accompanied by overproduction of heme-precursor molecules, specifically delta-ALA and porphobilinogen [16, 17]. In LP, the metal directly inhibits aminolevulinic acid dehydratase production. The result is an overproduction of aminolevulinic acid with normal porphobilinogen levels, reflecting the impaired conversion of the delta-ALA to porphobilinogen (Figure 1). In acute intermittent porphyria, porphyrin is not altered, as delta-ALA and porphobilinogen are porphyrin precursors. According to our case report, the diagnosis of acute porphyria based on a positive urine porphyrin test result was incorrect. Testing for heme precursors such as delta-ALA and porphobilinogen in urine is the key to the differential diagnosis between LP and acute porphyria. In acute porphyria, urine delta-ALA and urine porphobilinogen levels are elevated [18]. In our patient, the urine study revealed an elevated delta-ALA level (81.8 mg/L) but normal porphobilinogen level. Therefore, LP was considered as the correct diagnosis. The definitive diagnosis of LP was confirmed based on the high blood lead level.

Since antiquity, the most common source of LP is occupational exposure. Currently, occupational lead exposure has declined overall worldwide owing to the regulation of industrial practices. In recent years, LP is caused by nonoccupational exposure such as using herbal medicines or drugs, which is increasingly reported [1, 2, 19]. In our case, the Chinese herbal medicine that the patient had taken as therapy for urinary difficulty was finally considered as the most probable source of the lead exposure. Unfortunately, our patient did not report any history of herbal medicine use during the initial evaluation in the ED. This led to the misdiagnosis. Therefore, physicians should be aware that, with the increasing practice of self-medication with drugs from uncontrolled sources, the risk of drug-induced poisoning could increase in the future. Thus, routine and careful medical history taking should be included in obtaining comprehensive details about herbal medicine use.

Lead toxicity is reversible if diagnosed early by removal of the sources of exposure and early chelation therapy. LP may be fatal if the diagnosis is delayed or untreated [4–8, 19, 20]. Chelation therapy is needed in more severe cases based on whole-blood lead levels and the presence of symptoms [4, 5, 21]. Symptoms such as abdominal pain, subtle mood change, headache, irritability, or neuropathy warrant treatment with parenteral chelation. Chelation therapy is recommended when blood lead levels exceed 80 *µ*g/dL in asymptomatic patients and 50 *µ*g/dL in symptomatic patients [19, 21]. Chelation agents include the oral agent succimer, the intramuscular agent dimercaprol, and the intravenous agent CaEDTA [19–21]. Patients requiring parenteral therapy should be admitted to the hospital. Adequate hydration and urinary output are also important. Chelation therapy is usually stopped when symptoms resolve or when blood lead levels return to premorbid levels. A further course of chelation may be required if blood lead levels are still over 50 *µ*g/dL after the end of the initial course of treatment. An EDTA lead-mobilization test can be used to determine whether chelating therapy is necessary. This test measures the urinary excretion of lead over 8 h after CaEDTA is given. The amount of lead excreted per milligram of CaEDTA given is calculated. If the ratio is above 0.6, chelating therapy is indicated. A 24-h urinary lead excretion and urinary ALA may also be used to adjust the duration of therapy [22]. Our patient had a high blood lead level (62.8 *µ*g/dL) and abdominal colic. He was treated with parenteral chelation (CaEDTA) for 5 days as recommended by a toxicologist, with a rapid improvement in symptoms. In our case, the decision to adjust or cease chelation therapy was according to a toxicologist's expert advice and the resolution of the patient's symptoms. No relapse occurred thereafter and during the 1-year follow-up

## 4. Conclusion

This report provides physicians with the awareness that LP can be misdiagnosed as NSAP and acute porphyria. Testing for heme precursors in urine is the key to the differential diagnosis between LP and acute porphyria. A detailed traditional medical history taking is important for the diagnosis of LP. Therefore, each element of the triad of unexplained recurrent abdominal pain, anemia with basophilic stippling, and history of traditional medical intake should be considered in the diagnosis of lead poisoning.

## Figures and Tables

**Table 1:** The abnormal laboratory results obtained on admission and discharge of patient.

Laboratory data	On day 1 of admission	On day 19 after chelation therapy	Normal range
Hemoglobin	9.4 g/dL	11.5 g/dL	13.5–17.5
Hematocrit	27.9%	42%	41–53%
AST	57 U/L	32 U/L	0–37 U/L
ALT	87 U/L	40 U/L	0–40 U/L
Blood lead level	62.8 g/dL	31 g/dL	<40 *μ*g/dL
Urine delta-ALA	81.8 mg/L	nil	<7 mg/L/24 hr
Urine PBG	1.0 mg/L	nil	<4 mg/L/24 hr

ALT = alanine aminotransferase; AST = aspartate aminotransferase; PBG = porphobilinogen; ALA = aminolevulinic acid.

## References

[1] Pagliuca A., Mufti G. J., Baldwin D., Lestas A. N., Wallis R. M., Bellingham A. J. (1990). Lead poisoning: Clinical, biochemical, and haematological aspects of a recent outbreak. *Journal of Clinical Pathology*.

[2] Coyle P., Kosnett M. J., Hipkins K. (2005). Severe lead poisoning in the plastics industry: A report of three cases. *American Journal of Industrial Medicine*.

[3] Frith D., Yeung K., Thrush S., Hunt B. J., Hubbard J. G. H. (2005). Lead poisoning - A differential diagnosis for abdominal pain. *Lancet*.

[4] Sood A., Midha V., Sood N. (2002). Pain in abdomen-do not forget lead poisoning. *Indian Journal of Gastroenterology*.

[5] Chang S. H., Yoon S. B., Lee J. W., Lee D. J. (2013). What caused hemolytic anemia and colicky abdominal pain? Lead!. *Korean Journal of Internal Medicine*.

[6] Aktar A. J., Funneye A. S., Akanno J. (2003). Gunshot-induced plumbism in an adult male. *Journal of the National Medical Association*.

[7] Karri S. K., Saper R. B., Kales S. N. (2008). Lead encephalopathy due to traditional medicines. *Current Drug Safety*.

[8] Patrick L. (2006). Lead toxicity, a review of the literature. Part 1: exposure, evaluation, and treatment. *Alternative Medicine Review*.

[9] Beattie A. D., Briggs J. D., Canavan J. S., Doyle D., Mullin P. J., Watson A. A. (1975). Acute lead poisoning: five cases resulting from self-injection of lead and opium. *The Quarterly Journal of Medicine*.

[10] Mohammadi S., Mehrparvar A. H., Aghilinejad M. (2008). Appendectomy due to lead poisoning: A case-report. *Journal of Occupational Medicine and Toxicology*.

[11] Shiri R., Ansari M., Ranta M., Falah-Hassani K. (2007). Lead poisoning and recurrent abdominal pain. *Industrial Health*.

[12] Bruce S. G., Arbieva Z., Igor M. G. (2012). Analysis of lead toxicity in human cells. *BMC Genomics*.

[13] Smitherman J., Harber P. (1991). A case of mistaken identity: Herbal medicine as a cause of lead toxicity. *American Journal of Industrial Medicine*.

[14] Senut M.-C., Cingolani P., Sen A. (2012). Epigenetics of early-life lead exposure and effects on brain development. *Epigenomics*.

[15] McEwen J., Paterson C. (1972). Drugs and false-positive screening tests for porphyria. *British Medical Journal*.

[16] Lamon J. M. (1977). Clinical aspects of porphyrin in porphyrin measurement , other than lead poisoning. *Clinical Chemistry*.

[17] Venkatesh T. (2013). Editorial role of a clinical biochemist in evaluating the impact of lead poisoning. *Indian Journal of Clinical Biochemistry*.

[18] Martin C. J., Werntz C. L., Ducatman A. M. (2004). The interpretation of zinc protoporphyrin changes in lead intoxication: A case report and review of the literature. *Occupational Medicine*.

[19] Silbergeld E. K. (1995). The international dimensions of lead exposure. *International Journal of Occupational and Environmental Health*.

[20] Gidlow D. A. (2004). Lead toxicity. *Occupational Medicine*.

[21] Alessio L., Cortesi I., Materzanini P., Barenghi M. (2000). One century of studies on lead poisoning in papers published in La Medicina del Lavoro. *American Journal of Industrial Medicine*.

[22] Markowitz M. E., Rosen J. F. (1984). Assessment of lead stores in children: Validation of an 8-hour CaNa2EDTA provocative test. *The Journal of Pediatrics*.

